# ICU Admission-Related Factors Affecting the Duration of Mechanical Ventilation After Elective Cardiac Surgery—Retrospective Cohort Study from a Tertiary Center in Croatia

**DOI:** 10.3390/medicina61101778

**Published:** 2025-10-01

**Authors:** Darko Kristović, Verica Mikecin, Ivana Presečki, Zrinka Šafarić Oremuš, Nataša Sojčić, Ivan Gospić, Hrvoje Lasić, Sanja Sakan, Danijela Kralj Husajna, Nikola Bradić, Jasminka Peršec, Andrej Šribar

**Affiliations:** 1 Clinical Department of Anesthesiology, Resuscitation and Intensive Care Medicine, University Hospital Dubrava, 10000 Zagreb, Croatia; dkanezg@gmail.com (D.K.); zsafarico@gmail.com (Z.Š.O.); daca1310@gmail.com (D.K.H.); nbradic@unin.hr (N.B.); 2 LKH Universitätsklinikum Graz, 8036 Graz, Austria; 3 Department of Nursing, University North, 42000 Varaždin, Croatia; 4 University of Zagreb, School of Dental Medicine, 10000 Zagreb, Croatia

**Keywords:** mechanical ventilation, ICU, cardiac surgery, risk assessment

## Abstract

*Background and Objectives*: Enhancing recovery after cardiac surgery involves minimally invasive procedures, early extubation/mobilization, and swift discharge. While mechanical ventilation is often essential post-operation, prolonged invasive ventilation (IMV) increases mortality risk. Duration is influenced by patient factors (age and comorbidities), surgical complexity, and complications. Prognostic scores like EuroSCORE II, sequential organ failure assessment (SOFA), the Charlson Comorbidity Index (CCI), and the vasoactive–inotropic score (VIS) help to predict ventilation needs. The aim of this study is to analyze the effect of pre-/post-operation factors and procedure type as predictors of ventilation time. *Materials and Methods*: This is a retrospective cohort observational study analyzing factors affecting the duration of postoperative mechanical ventilation in elective cardiac surgical patients treated between 1 January and 31 December 2024 in a tertiary center in continental Croatia. Patients were stratified into two groups according to the duration of IMV: normal (first three quartiles) and prolonged (upper quartile). In total, 493 elective cardiac surgical patients operated on under general endotracheal anesthesia with sternotomy or mini-sternotomy were admitted postoperatively to the cardiovascular ICU and mechanically ventilated during the observed period, and 463 patients were included in the final analysis after the exclusion criteria had been applied. *Results*: The mean age was 64.7 ± 9.8 years, and 28.7% of the cohort were females while 71.3% were males. The median Charlton Comorbidity Index was 4 (IQR 3–5), the VIS was 2 (IQR 0–3), the SOFA score at ICU admission was 5 (IQR 3–6), and the adjusted SOFA score was 3 (IQR 2–4). In the multivariate logistic regression model, a significant effect of female sex (OR 1.98), age (OR 1.05), VIS (OR 1.05), and history of previous cardiac surgery (OR 6.67) on the duration of mechanical ventilation was observed. In the time-to-extubation multivariate analysis, there was a significant effect of re-do surgery (HR 3.70), corrected SOFA score (HR 1.14), and VIS (HR 1.05) on the duration of mechanical ventilation. There was no significant effect of the type of surgery (CABG, aorta, aortic valve, mitral/tricuspid valve, or other) or the amount of chest tube drainage on the duration of mechanical ventilation. *Conclusions*: A history of previous cardiac surgery and the vasoactive–inotropic score during the first 24 postoperative hours in the ICU are the strongest predictors of the duration of mechanical ventilation after elective cardiac surgery, with a statistically significant effect present in both the logistic regression model and hazard ratio analysis. Further analyses with more variables are warranted in the future to refine the prognostic model.

## 1. Introduction

One primary strategy for enhancing recovery after cardiac surgery—by reducing perioperative morbidity and mortality and improving quality of life—is to prioritize minimally invasive procedures (where applicable), early extubation, early mobilization, and prompt ICU and hospital discharge [1].

Mechanical ventilation provides critical postoperative support for most cardiac surgery patients, facilitating the recovery of cardiac and respiratory function and often continuing beyond the operating room. While non-invasive ventilation is typically used for less complex cases and weaned within 12–24 h, invasive mechanical ventilation (IMV) is inevitable for a majority of patients. The need for IMV is associated with numerous risk factors. Preoperative factors include poor cardiac function, history of myocardial infarction, atrial fibrillation, advanced age, smoking, chronic obstructive pulmonary disease (COPD), pulmonary hypertension, chronic kidney disease, and obesity. Intraoperative risk factors encompass prolonged cardiopulmonary bypass and aortic cross-clamp times, extended surgery duration, complex procedures, sustained hypothermia, excessive blood loss, and transfusion. Finally, postoperative factors such as bleeding, delirium, acute kidney injury, respiratory failure, and pneumonia also contribute.

The duration of mechanical ventilation after elective cardiac surgery is influenced by multiple variables, including patient characteristics, surgical complexity, perioperative management (including ventilation strategies [2,3]), and the incidence of postoperative complications [4]. Consequently, various prognostic scoring systems—derived from preexisting conditions and/or acute clinical and laboratory features—are used for critically ill patients.

According to a bibliometric analysis by Zhang et al. [5], which examined 1969 articles, there is a significantly increasing trend in publications focusing on mechanical ventilation for ICU patients after cardiac surgery and its associated prognostic factors. These factors critically influence ventilation duration and the probability of successful weaning. This focus is warranted, as prolonged invasive mechanical ventilation (IMV) affects 10–20% of cardiac surgery patients and is linked to a 50% increase in surgery-related mortality. Among the tools used to predict such outcomes, the EuroSCORE II is common and effective, predicting both postoperative mortality (AUC 0.791) and the risk of prolonged mechanical ventilation (AUC 0.711) [5].

Another key tool is the sequential organ failure assessment (SOFA) score, a composite measure of organ dysfunction. While primarily used in septic patients, its utility as a predictor of ICU morbidity and mortality has been validated in various critically ill populations, including cardiac surgery patients [6,7], though some studies suggest it is less effective than APACHE IV or SAPS II [8]. Despite this, its simplicity ensures its widespread use in ICUs globally. Finally, the Charlson Comorbidity Index, developed in 1987 [9], is a widely used prognostic tool for determining short- and long-term mortality. Although most evident in predicting long-term outcomes, its application has expanded to assessing ICU mortality [10].

The vasoactive–inotropic score (VIS) is a quantitative tool used in intensive care to objectively measure the level of cardiovascular support a patient requires, particularly after cardiac surgery. By aggregating the doses of multiple vasoactive and inotropic medications into a single value weighted for each agent’s potency, the VIS provides a composite measure of cardiovascular dysfunction. Its utility as a prognostic indicator for postoperative outcomes is well-validated [11]. This is underscored by a systematic review and meta-analysis by Sun et al., which, after sensitivity analysis for study heterogeneity, found a significant association between VIS and prolonged mechanical ventilation, with an odds ratio (OR) of 5.20 [12].

Building on this evidence, the present study aimed to determine the prognostic effect of specific patient characteristics on the duration of postoperative mechanical ventilation following elective cardiac surgery. The analyzed variables included preoperative factors (age, sex, Charlson Comorbidity Index, and history of previous cardiac surgery), postoperative factors (chest tube drainage, SOFA score, and VIS), and the type of procedure performed. Unlike most studies that combine elective and urgent/emergent cases, this analysis focused exclusively on an elective patient population. This deliberate focus was chosen to refine risk assessment and identify strategies to reduce mechanical ventilation duration and ICU stay in a more homogeneous cohort.

## 2. Materials and Methods

### 2.1. Design

This is a retrospective, cohort observational study analyzing factors affecting the duration of postoperative mechanical ventilation in elective cardiac surgical patients treated between 1 January and 31 December 2024, at a tertiary center in continental Croatia.

### 2.2. Participants

Inclusion criteria: Patients over 18 years of age who underwent elective cardiac surgery under general endotracheal anesthesia using sternotomy or mini-sternotomy.

Exclusion criteria: Revision surgery performed within the first 24 h after ICU admission due to bleeding or other complications requiring reoperation; death during ICU stay while still intubated.

**Figure 1:**
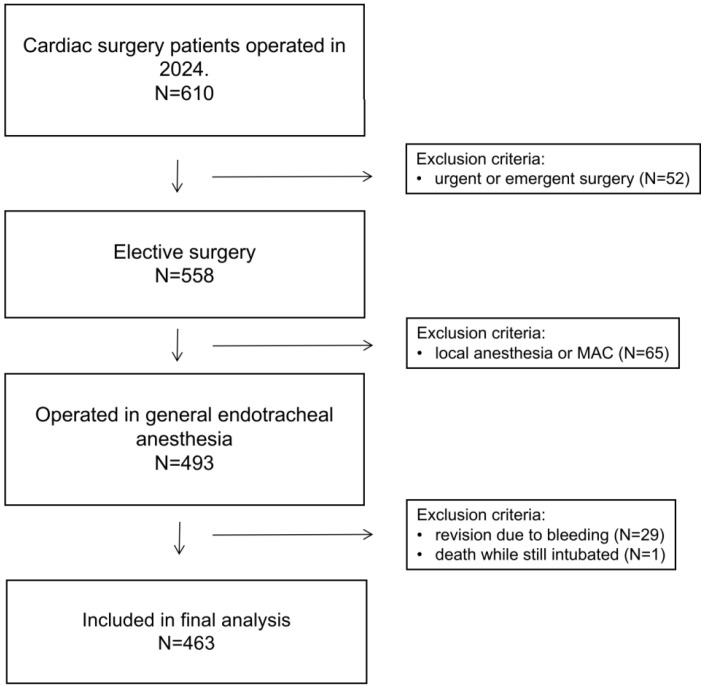
Patient selection flow diagram.

The participant selection flowchart is displayed in Figure 1.

### 2.3. Outcome Measure

Duration of mechanical ventilation with successful weaning (characterized by extubation without the subsequent need for reintubation or non-invasive ventilation due to respiratory failure). The upper quartile (10 h) was chosen as the cut-off between prolonged and normal duration of mechanical ventilation.

### 2.4. Weaning and Extubation Criteria

While no formal institutional protocol for weaning and extubation was in place, the standard practice in the ICU involved a systematic approach. Analgosedation (typically intravenous morphine hydrochloride) was routinely discontinued once the patient achieved a core temperature of 36 °C and chest tube drainage was less than 50 mL/h. The weaning process then commenced with a transition from a controlled ventilator mode to a pressure support mode. Following this, an awake and responsive patient would undergo a spontaneous breathing trial, conducted either with a T-piece or on the ventilator. The final step was extubation, which was performed if arterial blood gas values showed no significant hypoxemia or hypercarbia relative to the patient’s preoperative baseline.

### 2.5. Data Collection

Patient data were extracted from the hospital’s electronic medical record system (In2, Zagreb, Croatia) and compiled into a spreadsheet. Within this dataset, anonymized participants were listed as rows, with various patient- and procedure-related variables as columns. The recorded variables included age, sex, the highest vasoactive–inotropic score (VIS) [12] within the first 24 h of ICU stay, the Charlson Comorbidity Index (CCI) [9,10], the sequential organ failure assessment (SOFA) score at ICU admission, an adjusted SOFA score (excluding the cardiovascular component), and the type of surgery. Surgical procedures were categorized into the following groups: coronary artery bypass grafting (CABG), aortic valve replacement/repair, mitral valve replacement/repair, ascending aorta/aortic arch procedures, and other procedures.

### 2.6. Clinical Scoring Tools

CCI and SOFA scores are routinely collected at ICU admission, while VIS is calculated as the highest value recorded during the first 24 h of ICU stay.

Variables used to calculate the above-mentioned scores are listed in Table 1.

### 2.7. Statistical Analysis

Continuous data are presented as the mean ± standard deviation (SD) for normally distributed variables or as the median with interquartile range (IQR) for non-normally distributed variables; normality was assessed using the Shapiro–Wilk test. Categorical data are summarized as frequencies and percentages.

Group comparisons were conducted using appropriate tests based on data distribution and type. For continuous variables, differences between two groups were analyzed with the independent samples Student’s *t*-test (normal distribution) or the Mann–Whitney U test (non-normal distribution). Comparisons across three or more groups used one-way analysis of variance (ANOVA) or the Kruskal–Wallis test, with post hoc pairwise comparisons performed using the Holm–Šidak test (following ANOVA) or the Dwass–Steel–Critchlow–Fligner test (following Kruskal–Wallis). For categorical variables, the χ^2^ test or Fisher’s exact test (for 2 × 2 tables) was applied.

Based on the interquartile range of ventilation duration, patients were dichotomized into two groups: a “normal” duration group (first three quartiles) and a “prolonged” duration group (fourth quartile). The univariate association of each predictor variable (e.g., age, sex, VIS, SOFA scores, surgery type) with this outcome was assessed using the Mann–Whitney U or χ^2^ test. Variables with a univariate *p*-value < 0.1 were included in a subsequent multivariate logistic regression model.

Prior to finalizing the logistic regression model, variables were checked for multicollinearity using the variance inflation factor (VIF); any variable with a VIF > 2.5 was excluded. The model’s goodness-of-fit was evaluated with the Hosmer–Lemeshow test. The impact of categorical variables on ventilation duration over time was further analyzed with Kaplan–Meier curves and the log-rank test.

Due to the statistical software’s output format, which frames the outcome as a decrease in ventilation time, all odds ratios (OR) and hazard ratios (HR) reported in the results are presented as inverted values (1/OR, 1/HR). Thus, an increase in ventilation duration is associated with a decrease in the reported ratio value.

All analyses were performed using jamovi (version 2.6.44) with the ClinicoPath (v0.0.2) and Survival (v3.8.3) modules [13,14,15]. A two-sided *p*-value < 0.05 was considered statistically significant.

## 3. Results

### 3.1. Patient Characteristics

After the exclusion of patients who underwent revision on the day of surgery due to postoperative bleeding and/or died during ICU stay while still intubated, 463 patients were included in the final analysis.

**Figure 2:**
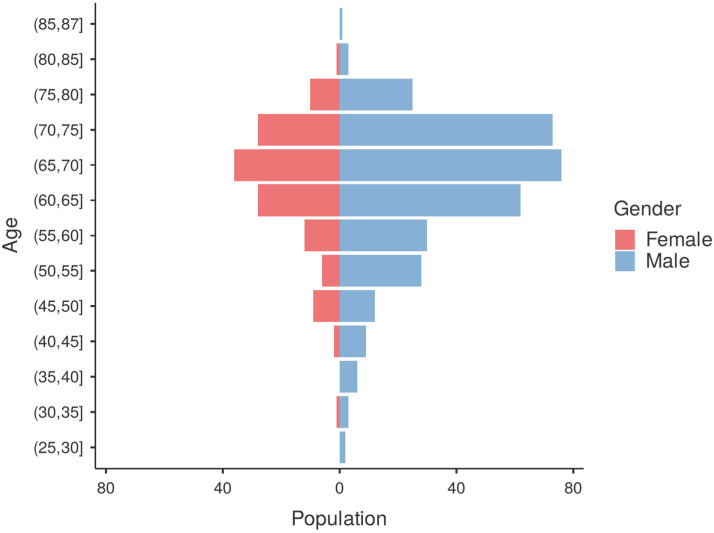
Distribution of age groups between males and females.

The study population had a mean age of 64.7 ± 9.8 years and comprised 330 males (71.3%) and 133 females (28.7%); the age distribution by sex is presented in Figure 2. The median Charlson Comorbidity Index was 4 (IQR 3–5). At ICU admission, the median vasoactive–inotropic score (VIS) was 2 (IQR 0–3), and the median sequential organ failure assessment (SOFA) score was 5 (IQR 3–6). The adjusted SOFA score (excluding the cardiovascular component) was 3 (IQR 2–4).

### 3.2. Factors Affecting the Duration of Mechanical Ventilation

**Figure 3:**
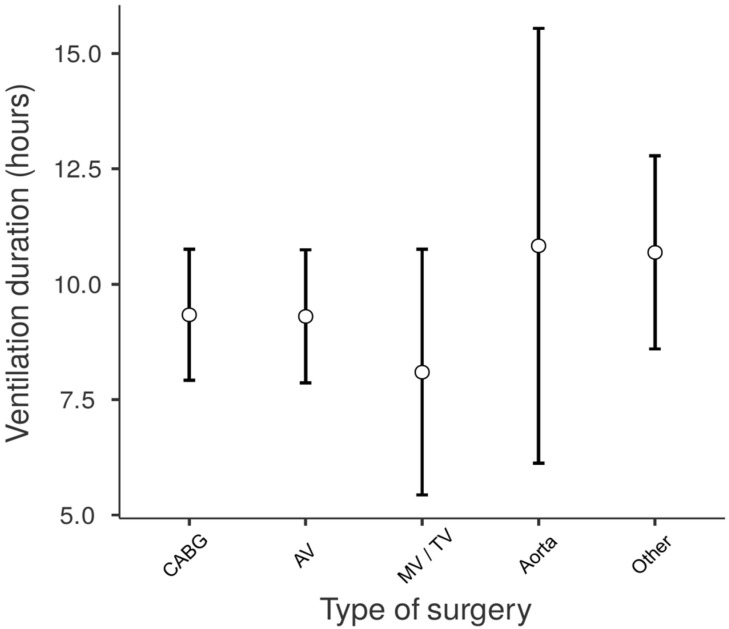
Differences in duration of mechanical ventilation between various types of surgical procedures.

The median duration of mechanical ventilation was 8 h (IQR 6–10). Based on this distribution, patients with a duration exceeding 10 h (the upper quartile) were classified as having prolonged mechanical ventilation. The type and duration of ventilation across different surgical procedures are detailed in Table 2. The type of procedure itself did not have a significant effect on ventilation duration (*p* = 0.496, ε^2^ = 0.07; Figure 3). This was also true within the CABG subgroup, where no significant difference was found between patients who underwent off-pump surgery (*N* = 52, median 7 h, IQR 6–9.5) and those who underwent surgery with cardiopulmonary bypass (CPB) (*N* = 113, median 8 h, IQR 6–10; *p* = 0.948).

Furthermore, no significant differences were observed between the prolonged and non-prolonged ventilation groups regarding the amount of chest tube drainage within the first 24 h (280 mL [IQR 200–400] vs. 250 mL [IQR 196–400], respectively; *p* = 0.72). This lack of significance was present even before the exclusion of patients who underwent surgical revision for bleeding (290 mL [IQR 200–400] vs. 300 mL [IQR 200–604], respectively; *p* = 0.056).

The correlation coefficients between continuous variables and the duration of mechanical ventilation are presented in Table 3.

### 3.3. Prognostic Factors Determining the Duration of Mechanical Ventilation—Logistic Regression Model

A multivariate logistic regression model was constructed using the following variables: patient sex, history of previous cardiac surgery, Charlson Comorbidity Index (CCI), SOFA score, and vasoactive–inotropic score (VIS). Initial analysis revealed multicollinearity, indicated by an elevated variance inflation factor (VIF) between the SOFA score and the VIS. This was expected, as the cardiovascular component is the primary contributor to the SOFA score in this patient population, followed by the respiratory component. To address this, the corrected SOFA score (excluding the cardiovascular component) was substituted in the final model. Both univariate and multivariate odds ratios for all variables are presented in Table 4.

A multivariate logistic regression analysis, stratified by procedure type, revealed that significant predictors of mechanical ventilation duration varied across surgical groups. For patients undergoing aortic valve procedures, significant variables included increased age (OR 0.89, 95% CI 0.82–0.95, *p* = 0.001), a history of previous cardiac surgery (OR 0.02, 95% CI 0.00–0.25, *p* = 0.005), and a higher vasoactive–inotropic score (VIS) (OR 0.90, 95% CI 0.84–0.96, *p* = 0.001). In the coronary artery bypass grafting (CABG) group, male sex was a significant predictor (OR 3.29, 95% CI 1.41–7.71, *p* = 0.006). For mitral and/or tricuspid procedures, a higher VIS was a significant factor (OR 0.84, 95% CI 0.72–0.95, *p* = 0.016). No statistically significant predictors were identified for patients undergoing surgery on the ascending aorta or those in the “other procedures” category.

### 3.4. Prognostic Factors Determining the Duration of Mechanical Ventilation—Time-to-Event Analysis

A time-to-event analysis of the duration of mechanical ventilation was performed using the same variables. Following multivariate adjustment, only a history of previous cardiac surgery, the corrected SOFA score, and the VIS demonstrated a significant independent association with ventilation duration (Table 5).

When stratified by procedure type, distinct predictors emerged. For aortic valve procedures, significant variables were older age (HR 0.96, 95% CI 0.94–0.98, *p* = 0.001), a history of previous cardiac surgery (HR 0.21, 95% CI 0.07–0.61, *p* = 0.004), and a higher VIS (HR 0.96, 95% CI 0.93–0.98, *p* = 0.002). In the coronary surgery group, male sex (HR 1.68, 95% CI 1.14–2.49, *p* = 0.009) and older age (HR 0.97, 95% CI 0.95–0.99, *p* = 0.003) were significant. For mitral and/or tricuspid procedures, a higher VIS (HR 0.92, 95% CI 0.88–0.97, *p* < 0.001) and older age (HR 0.94, 95% CI 0.90–0.97, *p* = 0.001) were associated with longer ventilation. Among patients undergoing “other procedures,” a higher corrected SOFA score (HR 0.84, 95% CI 0.74–0.96, *p* = 0.011) and a higher VIS (HR 0.94, 95% CI 0.89–0.98, *p* = 0.005) were significant predictors. No significant variables were identified for surgery on the ascending aorta.

**Figure 4:**
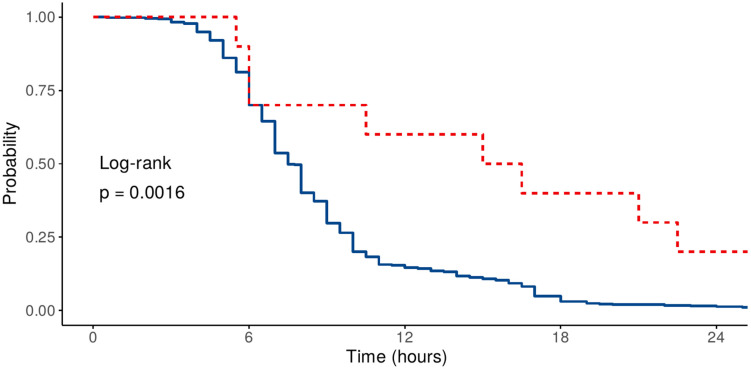
Kaplan–Meier curves depicting proportion of ventilated patients at various time points (full line—no previous surgery; dotted line—previous surgery).

**Figure 5:**
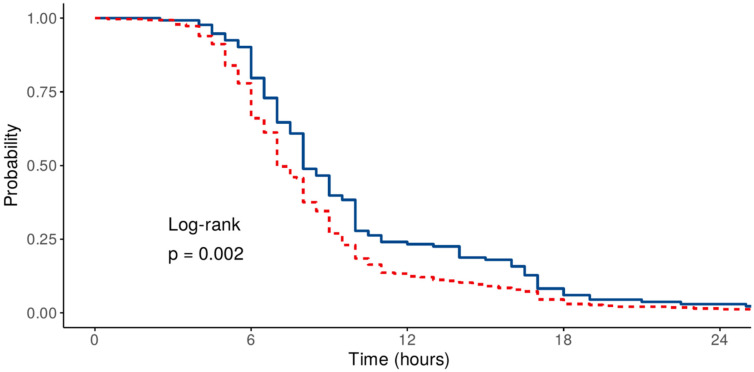
Kaplan–Meier curves depicting proportion of ventilated patients at various time points (full line—females; dotted line—males).

Kaplan–Meier curves illustrating the proportion of patients remaining on mechanical ventilation over time are presented in Figure 4 and Figure 5, accompanied by univariate log-rank test results. A significant difference was observed for sex and history of previous cardiac surgery. In contrast, no significant difference was found when comparing groups by the type of surgical procedure (log-rank test *p* = 0.39).

## 4. Discussion

The demographic and clinical characteristics of the analyzed patient cohort are consistent with those reported in similar studies [5,7,11].

Correlation analysis revealed statistically significant, albeit weak, associations between the duration of mechanical ventilation and age, VIS, CCI, and SOFA scores. The modest correlation coefficients suggest these variables may have limited independent clinical relevance for predicting ventilation duration in this specific elective surgical population, particularly when compared to findings from studies including emergent cases [10,12]. This attenuated effect may also be influenced by the absence of standardized institutional weaning and extubation protocols, which introduces inter-clinician variability.

After multivariate adjustment, a history of previous cardiac surgery was the strongest predictor of prolonged mechanical ventilation. This expected finding aligns with the existing literature [16] and is attributable to the inherent technical complexity of reoperations. Dense adhesions prolong dissection, cardiopulmonary bypass (CPB), and operative times, which amplifies the systemic inflammatory response and end-organ injury [17]. These factors collectively contribute to higher postoperative VIS and SOFA scores. Furthermore, reoperations often address more complex pathology (e.g., prosthetic valve failure), compounding the risk. It is important to note that our cohort consisted solely of elective cases and excluded high-risk congenital repairs, which are known to significantly impact ventilation times [16].

Other significant, though less pronounced, predictors included a higher vasoactive–inotropic score (VIS), male sex, advanced age, and a higher Charlson Comorbidity Index (CCI). The VIS serves as a quantitative surrogate for post-CPB myocardial dysfunction. An elevated score reflects low cardiac output syndrome, which impedes weaning by promoting cardiogenic pulmonary edema and limiting the heart’s ability to tolerate the hemodynamic shifts of spontaneous breathing. Advanced age contributes through reduced pulmonary compliance, diminished cardiac reserve, and sarcopenia affecting respiratory muscle strength. The CCI captures the cumulative burden of comorbidities that impair physiological reserve, such as COPD, heart failure, and renal disease. While one study found only the age-adjusted CCI significant [10], our results indicate a correlation with the non-adjusted score as well. Interestingly, unlike recent studies [18], chest tube drainage in the first 24 h was not a significant predictor in our cohort.

The SOFA score provides a broader assessment of multi-organ dysfunction. Its predictive value typically stems from quantifying the systemic insult from CPB and identifying patients with limited physiological reserve. However, in our analysis, the SOFA score did not retain independent significance after multivariate adjustment.

The definition of prolonged mechanical ventilation (PMV) varies considerably. This study used a 10-h cut-off (the upper quartile), whereas a 24-h threshold is more common [18,19,20]. Although the 24-h definition would enhance comparability, it was unsuitable for our cohort as only nine patients required ventilation beyond 24 h, and statistical models using this threshold demonstrated a poor fit (Hosmer–Lemeshow test, *p* < 0.001). A time-to-event analysis confirmed that a history of previous cardiac surgery, the corrected SOFA score, and the VIS were significant multivariate predictors of ventilation duration.

This study has several limitations. First, variables such as EuroSCORE II, CPB time, and cross-clamp time were not available for analysis, as they are not routinely recorded in the ICU database. Merging separate surgical and ICU databases was not feasible due to incompatible formats and resource constraints. Second, the absence of a standardized weaning protocol introduces potential bias, as extubation decisions were subject to individual clinician judgment. Implementing automated closed-loop weaning systems could mitigate this [2,3,21]. Third, specific comorbidities (e.g., diabetes, COPD) were not analyzed individually; instead, comorbidity burden was assessed collectively using the CCI.

## 5. Conclusions

A history of previous cardiac surgery and the vasoactive–inotropic score (VIS) within the first 24 postoperative hours emerged as the strongest predictors of prolonged mechanical ventilation following elective cardiac surgery. The statistical significance of both factors was confirmed through multivariate logistic regression and hazard ratio analyses. Future studies incorporating a broader range of variables would be valuable to further refine this prognostic model.

## Figures and Tables

**Table 1:** Clinical scoring tools used to assess level of vasoactive/inotropic support, comorbidity burden, or acute organ system status.

	Clinical Scoring Tool		
	VIS	CCI	SOFA
Measured Parameters (Score):	Vasoactive or Inotropic Drugs	Comorbidity Burden	Acute Organ System Status/Degree of Failure
	10,000 × vasopressin dose (IU/kg/min)	Age: <50, 50–59, 60–69, 70–79, >80 years (0–4 pts)	Respiratory (paO_2_/FiO_2_): 0–4 pts
	100 × epinephrine or norepinephrine dose (μg/kg/min)	Myocardial infarction: no/yes (0/1 pts)	Cardiovascular (MAP or vasoactive drug dose): 0–4 pts
	50 × levosimendan dose (μg/kg/min)	Congestive heart failure: no/yes (0/1 pts)	Neurologic (Glasgow Coma Score): 0–4 pts
	25 × olprinone dose (μg/kg/min)	Peripheral vascular disease: no/yes (0/1 pts)	Hepatic (bilirubin): 0–4 pts
	20 × methylene blue dose (mg/kg/h)	Cerebrovascular disease: no/yes (0/1 pts)	Renal (creatinine or diuresis): 0–4 mL
	10 × milrinone or phenylephrine dose (μg/kg/min)	Dementia: no/yes (0/1 pts)	
	10 × terlipressin dose (μg/min)	Chronic pulmonary disease: no/yes (0/1 pts)	
	1 × Dobutamine, dopamine, or enoximone dose (μg/kg/min)	Connective tissue disease: no/yes (0/1 pts)	
	0.25 × angiotensin II dose (ng/kg/min)	Peptic ulcer disease: no/yes (0/1 pts)	
		Liver disease: no/mild/moderate to severe (0, 1, 3 pts)	
		Diabetes mellitus: no, uncomplicated, end-organ damage (0–2 pts)	
		Hemiplegia: no/yes (0/2 pts)	
		Moderate-to-severe CKD: no/yes (0/2 pts)	
		Solid tumor: none/localized/metastatic (0/2/6 pts)	
		Leukemia: no/yes (0/2 pts)	
		Lymphoma: no/yes (0/2 pts)	
		AIDS: no/yes (0/2 pts)	

**Table 2:** Surgical procedures and duration of mechanical ventilation.

Type of Surgery	Counts (%)	Duration of Mechanical Ventilation (Hours, Median, IQR)
**CABG**	165 (35.6%)	8 (6–10)
**Aortic valve**	160 (34.6%)	7.5 (6–10)
**Mitral (+/− tricuspid) valve**	47 (10.2%)	7 (5–9.25)
**Ascendent aorta or aortic arch**	15 (3.2%)	7 (5.5–13)
**Other**	76 (16.4%)	8 (6–10.6)

**Table 3:** Correlation levels between duration of ventilation and continuous recorded variables.

		Age	CCI	VIS	Corrected SOFA	SOFA
Duration of ventilation	Spearman’s rho	0.277	0.216	0.187	0.148	0.232
	*p*-value	<0.001	<0.001	<0.001	0.001	<0.001

**Table 4:** Odds ratio table (univariable and multivariable) of factors affecting the duration of mechanical ventilation.

		Prolonged Ventilation			
		Yes	No	OR (95% CI)—Univariable	OR (95% CI)—Multivariable
**Age**	Mean (SD)	68.1 (7.2)	63.3 (10.3)	0.94 (0.92–0.97, *p* < 0.001)	0.95 (0.92–0.98, *p* = 0.003)
**Sex**	F	51 (38.3)	82 (61.7)	-	-
	M	76 (23%)	254 (77%)	2.08 (1.35–3.21, *p* = 0.001)	1.98 (1.24–3.15, *p* = 0.004)
**Previous cardiac surgery**	No	120 (26.5%)	333 (73.5%)	-	-
	Yes	7 (70%)	3 (30%)	0.15 (0.03–0.57, *p* = 0.007)	0.15 (0.03–0.65, *p* = 0.015)
**Corrected SOFA**	Mean (SD)	3.4 (1.8)	3.0 (1.7)	0.85 (0.76–0.96, *p* = 0.009)	0.91 (0.80–1.03, *p* = 0.141)
**CCI**	Mean (SD)	4.6 (1.7)	3.6 (1.9)	0.75 (0.67–0.84, *p* < 0.001)	0.86 (0.75–1.00, *p* = 0.041)
**VIS**	Mean (SD)	5.4 (8.5)	2.7 (5.3)	0.94 (0.91–0.97, *p* < 0.001)	0.95 (0.91–0.98, *p* = 0.001)

OR of normal vs. prolonged ventilation, AIC = 494.1, C-statistic = 0.727, H&L = χ^2^(8) 3.73 (*p* = 0.880).

**Table 5:** Hazard ratio table (univariable and multivariable) of factors affecting the duration of mechanical ventilation.

			HR (95% CI)—Univariable	HR (95% CI)—Multivariable
**Age**	Mean (SD)	64.7 (9.8)	1.00 (0.98–1.02, *p* = 0.970)	0.99 (0.96–1.01, *p* = 0.326)
**Sex**	Female	133 (28.7)	-	-
	Male	330 (71.3)	1.05 (0.74–1.50, *p* = 0.787)	0.81 (0.56–1.19, *p* = 0.291)
**Previous cardiac surgery**	No	453 (97.8)	-	-
	Yes	10 (2.2)	0.42 (0.19–0.91, *p* = 0.028)	0.27 (0.11–0.64, *p* = 0.003)
**Corrected SOFA**	Mean (SD)	3.1 (1.7)	0.89 (0.81–0.98, *p* = 0.020)	0.88 (0.79–0.98, *p* = 0.017)
**CCI**	Mean (SD)	3.8 (1.9)	0.96 (0.86–1.06, *p* = 0.395)	1.02 (0.91–1.15, *p* = 0.708)
**VIS**	Mean (SD)	3.4 (6.4)	0.96 (0.94–0.99, *p* = 0.002)	0.95 (0.92–0.98, *p* < 0.001)

HR ratio decrease is associated with increased duration of mechanical ventilation; concordance = 0.660 (SE = 0.031), R-squared = 0.061 (maximum possible = 0.880), likelihood ratio test = 29.228 (df = 6, *p* = 0.000).

## Data Availability

The raw data supporting the conclusions of this article will be made available by the authors on request.
